# A 5000-Fold Increase in the Specificity of a Bacterial Phosphotriesterase for Malathion through Combinatorial Active Site Mutagenesis

**DOI:** 10.1371/journal.pone.0094177

**Published:** 2014-04-10

**Authors:** Tatheer Naqvi, Andrew C. Warden, Nigel French, Elena Sugrue, Paul D. Carr, Colin J. Jackson, Colin Scott

**Affiliations:** 1 Department of Environmental Sciences, COMSATS Institute of Information Technology, Abbottabad, Pakistan; 2 Ecosystem Sciences, Commonwealth Scientific and Industrial Research Organisation, Canberra, Australian Capital Territory, Australia; 3 Research School of Chemistry, Australian National University, Canberra, Australian Capital Territory, Australia; University of Canterbury, New Zealand

## Abstract

Phosphotriesterases (PTEs) have been isolated from a range of bacterial species, including *Agrobcaterium radiobacter* (PTE*_Ar_*), and are efficient enzymes with broad substrate ranges. The turnover rate of PTE_Ar_ for the common organophosphorous insecticide malathion is lower than expected based on its physical properties; principally the pk_a_ of its leaving group. In this study, we rationalise the turnover rate of PTE*_Ar_* for malathion using computational docking of the substrate into a high resolution crystal structure of the enzyme, suggesting that malathion is too large for the PTE*_Ar_* binding pocket. Protein engineering through combinatorial active site saturation testing (CASTing) was then used to increase the rate of malathion turnover. Variants from a CASTing library in which Ser308 and Tyr309 were mutated yielded variants with increased activity towards malathion. The most active PTE*_Ar_* variant carried Ser308Leu and Tyr309Ala substitutions, which resulted in a *ca.* 5000-fold increase in *k*_cat_/*K*_M_ for malathion. X-ray crystal structures for the PTE*_Ar_* Ser308Leu\Tyr309Ala variant demonstrate that the access to the binding pocket was enhanced by the replacement of the bulky Tyr309 residue with the smaller alanine residue.

## Introduction

The World Health Organization has estimated that there are >3,000,000 cases of pesticide poisonings annually, which result in approximately 200,000 deaths. Many of these cases are due to accidental or deliberate intoxication with neurotoxic organophosphate pesticides (OPs) [1].

**Figure 1:**
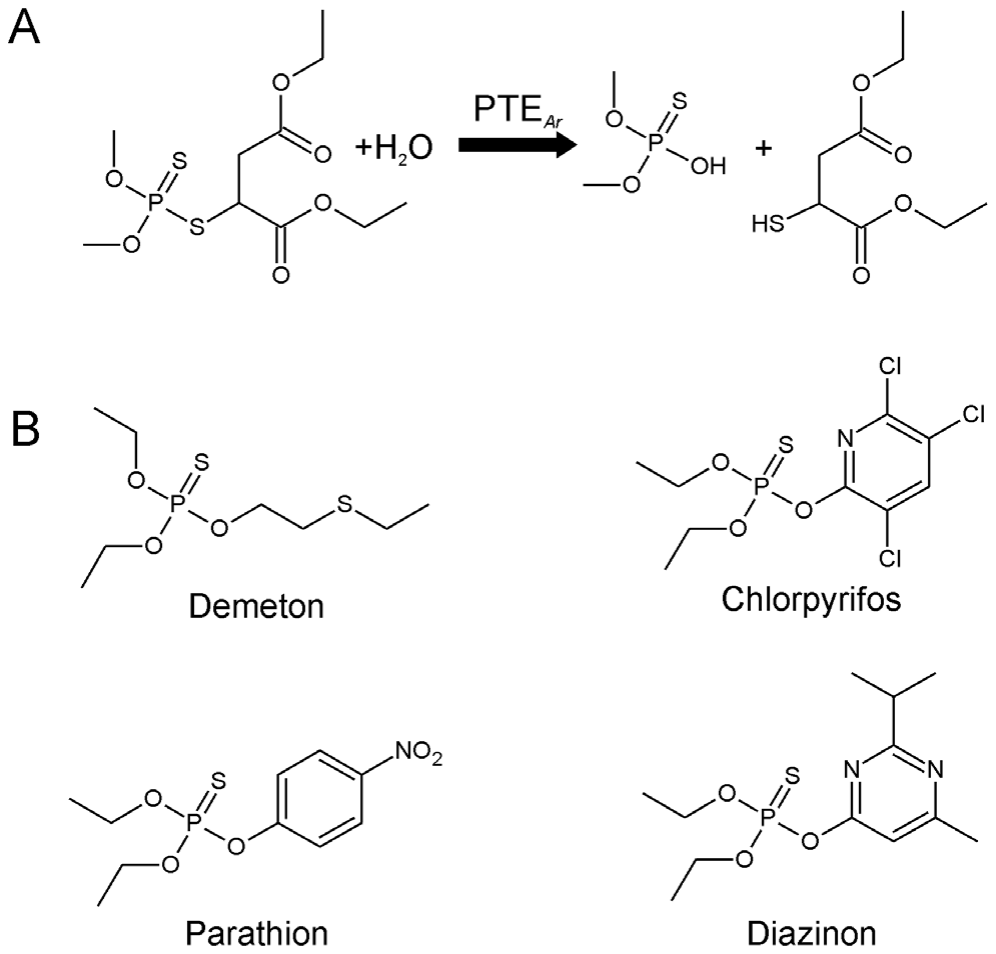
Hydrolytic activity of PTE*_Ar_*. A) Schematic showing the PTE*_Ar_*-mediated hydrolysis of malathion. B) Structure of the OP insecticides demeton, chlorpyrifos, parathion and diazinon.

OPs are potent cholinesterase inhibitors used extensively for the control of a variety of invertebrate pest species, but which also effect acute intoxication in humans [2]. The OPs share a phosphotriester structure, which is closely related to chemical warfare agents, such as VX and Sarin (Fig. 1). Enzymes that hydrolyse, and consequently detoxify, these phosphotriesters have been isolated from diverse origins [3], [4]. The best described of these enzymes are the bacterial phosphotriesterases (PTEs).

PTEs have been isolated from a range of bacterial species, including *Pseudomonas diminuta* (PTE*_Pd_*) and *Agrobacterium radiobacter* (PTE*_Ar_*) [5], [6]. They are binuclear metalloenzymes [7], highly efficient catalysts and display broad substrate specificities that include most phosphotriesters [5], [6], [8], [9]. PTEs have therefore been investigated for applications in environmental monitoring, pesticide decontamination, nerve agent detoxification and in clinical applications [9]–[15].

**Figure 2:**
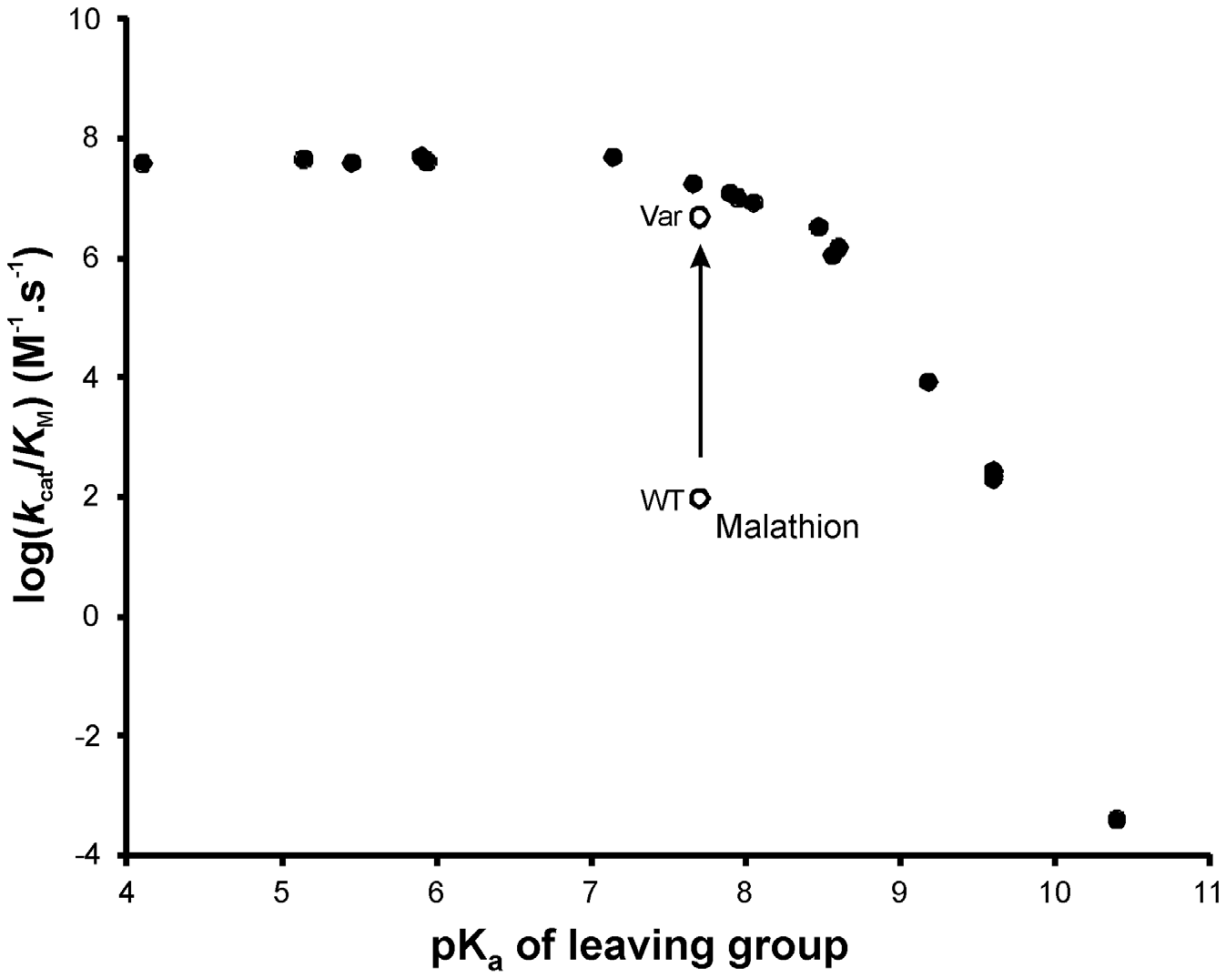
Brønsted plot of leaving group pKa values *vs* log(*k*_cat_/*K*_M_) for a range of substates. The p*K*_a_ values of the leaving groups 2,6-difluoro-4-nitrophenol; quinoxalin-2-ol; 2-fluoro-4-nitrophenol; 2-isopropyl-6-methylpyrimidin-4-ol; 3-fluoro-4-nitrophenol; 4-nitrophenol; 4-hydroxybenzaldehyde; 2,2-dichloroethylenol; 4-hydroxybenzonitrile; 1-(4-hydroxyphenol)ethanone; methyl 4-hydroxybenzoate; 4-hydroxybezamide; 3-chloro-7-hydroxy-4-methyl-2H-chromen-2-one; 2-(ethylthio)ethanethiol; 2-(diethylamino)ethanethiol; 2-(diisopropylamino)ethanethiol; and 4-(methoxymethyl)phenol plotted (left to right) against their log(*k*_cat_/*K*_M_) values. p*K*_a_ values were as published elsewhere [8], [17], [20] or as calculated using the SPARC online p*K*_a_ calculator (http://ibmlc2.chem.uga.edu/sparc/) [38]. The biphasic dependence of the enzyme on p*K*_a_ as described elsewhere [8], [20] is shown: the curve flattens below a p*K*_a_ of ∼8.0 and there is a linear dependence on p*K*_a_ at values below *ca*. 8.0.

The S_N_2 mechanism of PTE, using a metal-activated water as the nucleophile, is well documented [16]–[18]. This mechanism results in a biphasic dependence of the rate of hydrolysis upon the pK_a_ of the leaving group of the substrate (Fig. 2). For OPs with leaving groups that have pK_a_ values of ∼8.0 or lower the *k*_cat_/*K*_M_ for the reaction is near the diffusion limit, while at pK_a_ values of greater than ∼8.0 there is a linear relationship between pK_a_ and log (*k*_cat_/*K*_M_). Outliers to this trend have been documented, with their lower than expected turnover rates typically resulting from physical barriers to correct substrate binding, such as steric hindrance or non-productive binding [8], [19], [20].

The active site and substrate-binding pocket of the PTEs are also well defined, with an iron-zinc binuclear metal center co-ordinated by four histidine residues (His55, His57, His201 and His230) and aspartate (Asp301) and an unusual carbamylated lysine (Lys196), which bridges the center by co-coordinating both metals [7] (numbering for PTE*_Ar_*). The substrate-binding pocket is comprised of a number of largely hydrophobic residues: Gly60, Ser61, Ile106, Trp131, Phe132, Arg254, Tyr257, Leu271, Leu303, Phe306, Ser308 and Phe309 in PTE*_Ar_* [6], [17]–[20]. The large, hydrophobic substrate-binding pocket can accommodate the majority of anthropogenic phosphotriesters [3], [10], [19], including insecticides and nerve agents, and is responsible for the broad substrate range of the enzyme.

The efficiency of PTE against many such substrates, including chlorpyrifos, demeton-S, chlorfenvinphos, diisopropyl fluorophsphate and others, has been improved by directed (laboratory) evolution, rational design and incorporation of unnatural amino acids [18], [20]–[24]. However, there have been no reports of substantial improvements in the turnover rate of PTEs towards malathion, the most widely used OP insecticide in the US [25] with significant applications in the control of West Nile virus and fruit fly infestation [26], [27].

Herein we have used Combinatorial Active-Site Saturation Testing (CASTing) [28] to improve the efficiency of malathion turnover by reducing the rate limitation caused by steric hindrance of substrate binding.

## Methods and Materials

### Chemicals and reagents

All chemicals and reagents were obtained from Sigma-Aldrich. Malathion and its metabolites were of analytical grade and >99% pure. Synthetic oligonucleotides were obtained from GeneWorks (Australia). Restriction enzymes were obtained from New England Biolabs (Australia).

### Bacterial growth

*E. coli* Bl21 (λDE3) was used for screening and production of variant enzymes and was cultured in LB, on LB supplemented with 1.5% w/v agar (LBA) or in Terrific Broth [29]. The media were supplemented with 100 µg.mL^−1^ ampicillin as required.

### Site saturation libraries

pETMCS1-*opdA* was used as a template for the site saturation mutagenesis libraries. The method of Ho *et al*. [30] was used to make libraries of *opdA* mutants in which codons were replaced with the NNS degenerate codon. Mutant libraries targeted either single amino acid substitutions at position 106, 271 or 303 or two simultaneous amino acid substitutions at positions 60 and 61, 131 and 132, 254 and 257, 306 and 308, and 308 and 309. Each of the single substitution libraries encoded 32 variants and each of the double substitution libraries encoded 1024 variants. The library amplicons were cloned into pETMCSI [24] using *Nde*I and *Eco*RI. The diversity of mutations within each library was ascertained by sequencing plasmid DNA obtained from transformants prior to any laboratory selection.

### Screening for malathion hydrolase activity

For malathion hydrolase activity screening, competent BL21 (λDE3) were transformed with libraries cloned into pETMCS1, plated on LBA and incubated at 37 °C overnight. Ninety five transformants were screened from each library with single amino acid substitutions and 3070 transformants screened from each library with double amino acid substitutions; this resulted in each library being screened at ∼3× the diversity of the library. Transformants were transferred into two 96-well growth blocks (3 mL volume in each well), with each growth block containing 94 library transformants, one BL21 (λDE3) pETMCS1-*OpdA* colony (base-line control) and one BL21 (λDE3) colony (negative control).

Growth blocks were incubated at 37 °C over night then centrifuged at 5,300×g for 30 minutes to sediment the cultures. The pellets were resuspended and lysed in 3 mL Bugbuster solution (Novagen), according to the manufacturer's instructions. 40 µL of cell-free extract (CFE) from each well was transferred to a 96-well microtitre plate.

The rate of malathion hydrolysis by the CFEs was followed by measuring the rate of thiol group liberation (i.e. diethyl 2-mercaptosuccinate formation) using Ellman's reagent modified for use in 96-well microtitre plate format: 140 µl of 5 mM Ellman's reagent containing 20 mM malathion was added to 40 µl of CFE. The change in absorbance at 412 nm was measure for 30 min using a SpectraMax M2 spectrophotometer (Molecular Devices, CA).

Plasmids were obtained from transformants that possessed greater malathion hydrolase activity than the BL21 (λDE3) pETMCS1-*OpdA* control using a plasmid DNA purification kit (Macherey-Nagel, Germany), and the sequences of the *OpdA* mutants were obtained (Micromon, Melbourne).

### Protein expression, purification and crystallization

PTE*_Ar_* and variants were expressed in BL21 (λDE3) grown in TB supplemented with 100 µM CoCl_2_ at 30 °C for 48 hours. The cells were harvested after 48 hours by centrifugation (5,000×g for 15 min), pellets were then resuspended in 5 ml of 50 mM HEPES with 1 mM CoCl_2_ (pH 8) per gram of cells. Cells were lysed using an EmulsiflexC_3_ homogeniser (Avestin Inc., Germany) according to the manufacturer's instructions. Cell debris was removed by centrifuging at 20,000×g for 30 minutes and the supernatant was recovered.

PTE*_Ar_* and variants were purified as described elsewhere [17], [19]. All columns and chromatographic media were purchased from GE Healthcare. Protein concentration was determined using a nanodrop ND-1000 spectrophotometer (Thermofisher Scientific, Australia), assuming an extinction coefficient of 29,280 M^−1^.cm^−1^ [19]. Protein purity was monitored by using reducing pre-cast SDS-PAGE gels (NuSep, Australia) stained with Coomasie brilliant blue. PTE*_Ar_* was crystallised as previously described [7], [18].

### Crystal soaking and X-ray data collection

PTE*_Ar_* crystals were serially transferred to cryoprotectant solutions consisting of 40% PEG 3350 and 0.2 M NaNO3 with or without 2 mM malathion for 2 minutes before data collection. The crystals were flash-cooled to 100 K in a cryogenic nitrogen gas stream. Diffraction data were collected on a Marresearch marµX system, comprising a Xenocs Genix^3D^ Cu high flux generator and a mar345 image plate detector. All data reduction was performed using XDS and CCP4 [31], [32].

### Structure determination

Crystals were isomorphous to those previously solved (spacegroup *P*3121, a = 108.9, c = 62.4) [7], [18]; accordingly, this model was used to calculate the initial protein phases. REFMAC as implemented in the CCP4 suite of program [33], was employed for refinement. The structure and restrains for diethyl thiophosphate were taken from previous work [17]. Difference Fourier maps obtained from soaked PTE*_Ar_* crystals in the absence of bound substrate/products were obtained initially, followed by inclusion of the substrates/products in the models and real-space refinement against the positive density using restraints and the COOT program [34]. These were further refined using REFMAC. Ligand occupancy was adjusted until the B-factors of the ligands refined to values comparable to the interacting metal ions/amino acids.

### Enzyme assays

Rates of hydrolysis of malathion and demeton were measured by quantifying the formation of thiol groups using the modified Ellman's assay [35], essentially as described above, 140 µl of 5 mM Ellman's reagent containing malathion or demeton (0, 5, 10, 25, 50, 100, 150, 300, 400 and 600 µM) was added to 40 µl of 50 mM HEPES buffer containing 0.22–0.43 nM enzyme, as appropriate. Each assay was conducted in triplicate. An extinction co-efficient of 14,140 M^−1^.cm^−1^ [35] was used. Hydrolysis rates for diazinon, chlorpyrifos and parathion were obtained spectrophotometrically, as described elsewhere [10]. Rates were determined over 30 minutes. Values for *k*_cat_ and *K*_M_ were estimated using “Hyper32” hyperbolic regression software [20].

### Computational procedures

Docking of malathion to the PTE*_Ar_* native structure with a water molecule bridging the metal centres was performed using CDOCKER as implemented in Accelrys Discovery Studio [36]. The top 10 poses were taken from a run docking 40 orientations of 40 conformers of malathion with simulated annealing for each pose.

## Results and Discussion

### Malathion is a poor substrate for PTE_Ar_

PTE*_Ar_* has a *k*_cat_/*K*_M_ value for malathion of 4×10^2^ s^−1^.M^−1^. The pK_a_ of the leaving group of malathion (diethyl 2-mercaptosuccinate) is predicted to be 7.7, suggesting that malathion is turned over by PTE*_Ar_* with a *k*_cat_/*K*_M_ that is at least several order of magnitude lower than other phosphotriesters with leaving groups that have similar pK_a_ values (Fig. 2). This suggests that another parameter, such as substrate binding or formation of the Michaelis complex, may limit the rate of this reaction.

**Figure 3:**
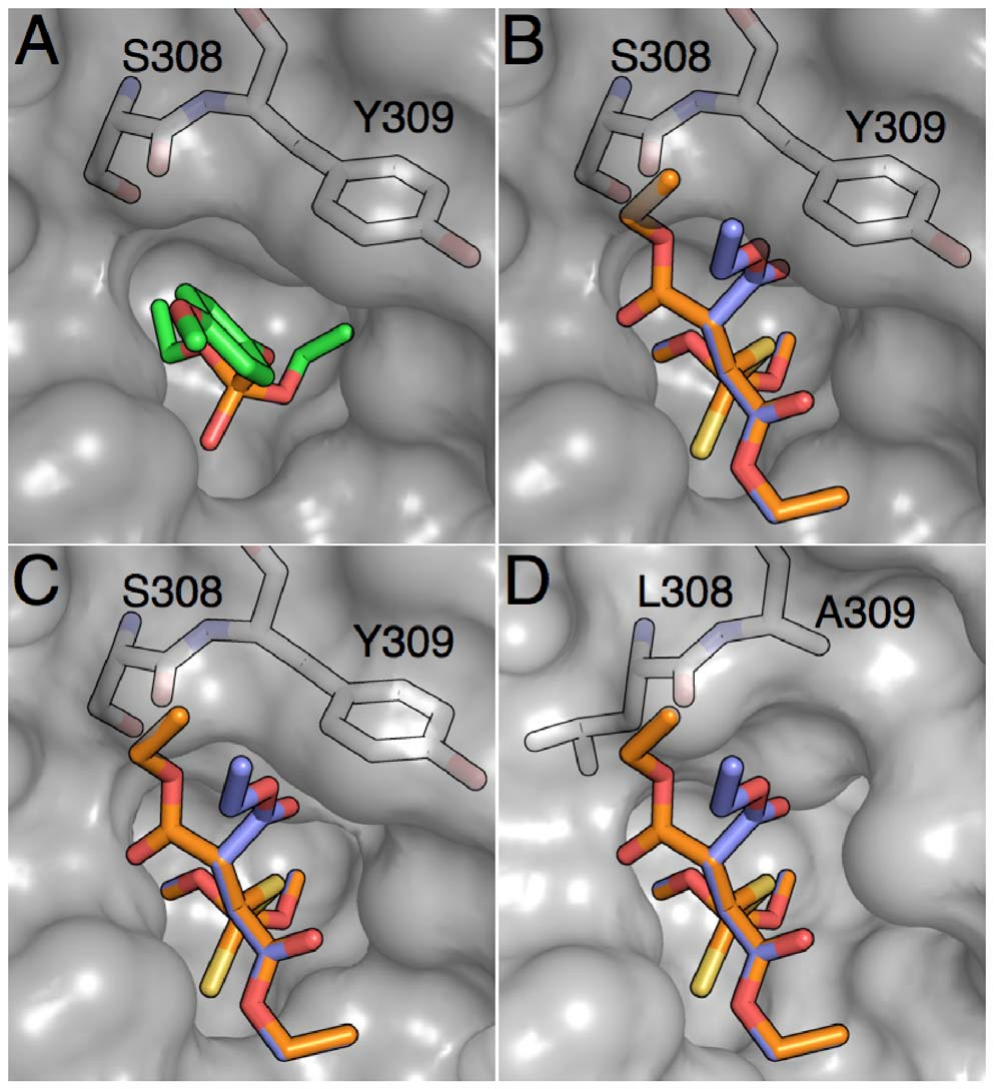
Docking of malathion into the crystal structures of wild-type PTE*_AR_* and PTE*_AR_* Ser308Leu/Tyr309Ala. A. The substrate-binding pocket of PTE*_Ar_* (2R1N) with bound substrate. The amino acids that were randomised in this experiment are labelled. B. Superimposed structures of malathion docked in the active site of PTE*_Ar_* in two different conformations. The branched leaving group of malathion results in steric clashes with the protein, primarily with Ser308 and Tyr309. C. Superimposed structures of malathion docked in the active site of the minor, open, conformation of PTE*_Ar_* in two different conformations. The steric clash with Tyr309 is lessened. D. Superimposed structures of malathion docked in the active site of the Ser309Leu/Tyr309Ala variant of PTE*_Ar_*. This shows that the Tyr309Ala mutation has opened the substrate-binding cavity, removing the steric hindrance to malathion binding in a productive conformation.

To rationalise the very low kinetic parameters of PTE*_Ar_* with malathion, we performed a series of computational docking procedures using the CDOCKER algorithm. CDOCKER has previously been verified crystallographically with PTE*_Ar_*, producing a docked pose that was essentially superimposable with a crystal structure of an PTE*_Ar_*-diethyl 4-methoxyphenyl phosphate complex [17](Fig. 3A). Forty orientations of forty conformers of malathion were docked, with simulated annealing for each pose. The results did not yield any poses that were productively bound with the substrate appropriately aligned for nucleophilic attack from the metal-ion coordinated nucleophile. To investigate further, we superimposed various conformers of malathion onto the substrate in a crystallographic PTE*_Ar_*:substrate complex (Fig. 3). This revealed that the substrate binding pocket was too small to accommodate the branched leaving group of malathion, with particular clashes near Ser308 and Tyr309 (Fig. 3B).

If malathion cannot bind to PTE*_Ar_*, then how can the low, but significant, turnover of this substrate be explained? Previous work was shown that PTE*_Ar_* fluctuates between open and closed conformations [37], and that alternative open conformations allowed the binding of another larger organophosphate, chlorfenvinfos [20]. When malathion is docked into this minor, open, conformation, it is clear that the steric clashes no longer prevent binding, i.e. a minor conformation appears to catalyze a non-native activity (Fig. 3C). This low-level promiscuous activity towards malathion, coupled with the relatively good leaving group of this substrate, suggests that significant increases in activity should be possible, provided the substrate binding site is sufficiently modified to favour formation of the Michaelis complex.

### CASTing for improved malathion hydrolysis

In accordance with the docking results, a semi-rational approach was used to improve the turnover of this substrate. CASTing was performed in the substrate-binding pocket of PTE*_Ar_*, with each of the residues that form the substrate-binding pocket included in at least one CASTing library. CASTing is an approach wherein analysis of an enzyme's 3D structure is used to identify groups of two or three amino acids from the binding-pocket, which are then randomized simultaneously to create relatively small libraries of mutants that can be screened with relatice ease. For PTE_Ar_, each CASTing library carried substitutions at one or two amino acid positions and therefore producing libraries of 32 or 1024 variants (using an NNS degeneracy). The libraries were: Gly60Xxx and Ser61Xxx, Ile106Xxx, Trp131Xxx and Phe132Xxx, Arg254Xxx and Tyr257Xxx, Leu271Xxx, Leu303Xxx, Phe306Xxx and Ser308Xxx, and Ser308Xxx and Tyr309Xxx. Cell-free extract from each library was screened for the rate of formation of free thiol (as a result of malathion hydrolysis; Fig. 1), with 95 or 3070 transformants screened per library depending upon the size of the library screened (i.e, screened at ∼3× the diversity of the library).

**Figure 4:**
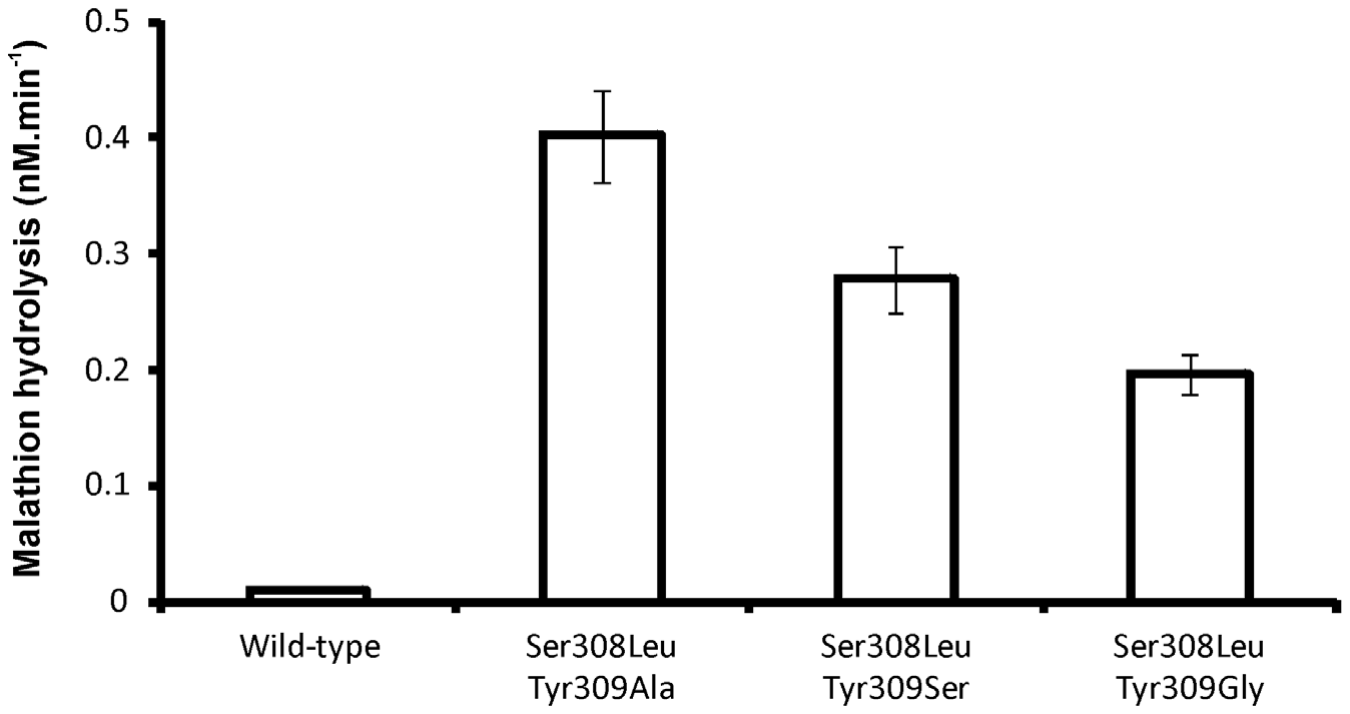
Screening for improved malathion hydrolase activity. The activity of three cell-free extracts from transformants isolated from the Ser308Xxx/Tyr309Xxx libraries are shown alongside that of the cell-free extract from a transformant obtained using the unmodified gene. The library transformants were subsequently shown to carry the Ser308Leu substitution and an Ala, Ser or Gly at 309. The data presented here are the averages of three independent assays, replicates varied by less than 10%.

Although the majority of transformants from all libraries retained some hydrolytic activity against malathion, only the library in which Ser308 and Tyr309 were targeted for mutagenesis provided variants with increased activity towards malathion compared with that of the wild-type enzyme (Fig 4). Variants with increased rates of malathion activity were found to carry a Ser308Leu substitution and a substitution of Tyr309 for Gly, Ser or Ala. Site-directed mutants of wild-type *opdA* were constructed that encoded only substitutions of Tyr309 for Gly, Ser or Ala; however, the expressed variants were insoluble.

The steady-state kinetic parameters of the most active variant (Ser308Leu, Tyr309Ala) were obtained and compared with those of the wild-type enzyme. There was a 2.7-fold decrease in the *K*_M_ for malathion (410 *vs.* 1,100 µM; Table 1) and a 2×10^4^-fold increase in *k*_cat_, (7.7×10^2^ *vs*. 3.9×10^−2^ s^−1^; Table 1), leading to a 5.4×10^4^-fold increase in the *k*_cat_/*K*_M_ (1.9×10^5^ *vs*. 3.5×10^1^ s^−1^.M^−1^; Table 1). Thus, amino acid substitutions at positions 308 and 309 alleviate the majority of the limitation on the rate of malathion hydrolysis by PTE*_Ar_*. Such large changes in turnover rates were not observed with a range of other OP insecticides including parathion (ca. 5-fold reduction in *k*_cat_/*K*_M_ value), *ca*. 2- and 9-fold increases in *k*_cat_/*K*_M_ values for chlorpyrifos and diazinon respectively, and no significant change in the rate of demeton turnover (Table 1). These data suggest that the specific interaction between PTE*_Ar_* and malathion *via* amino acids at positions 308 and 309 was responsible for the low turnover of malathion by the wild-type enzyme.

**Table 1:** Kinetic parameters of purified PTE*_Ar_* and most active variant against malathion for a range of OP insecticides.

Substrate		Wild Type		PTE*_Ar_*	Ser308Leu	/Tyr309Ala
	*k*_cat_(s^−1^)	*K*_M_(µM)	*k*_cat_/*K*_M_(s^−1^.M^−1^)	*k*_cat_(s^−1^)	*K*_M_(µM)	*k*_cat_/*K*_M_(s^−1^.M^−1^)
Malathion	3.9×10^−2^	1100	3.5×10^1^	7.6×10^2^	410	1.9×10^6^
	(2.1×10^−3^)	(97)		(3.3×10^1^)	(35)	
Parathion	6.2×10^3^	330	1.9×10^7^	9.1×10^2^	340	2.7×10^6^
	(9.3×10^1^)	(25)		(8.2×10^1^)	(31)	
Demeton	1.4×10^−2^	490	2.9×10^1^	1.1×10^−2^	490	2.2×10^1^
	(1.4×10^−3^)	(39)		(9.3×10^−4^)	(17)	
Diazinon	1.0×10^3^	480	2.1×10^6^	1.1×10^3^	430	2.5×10^7^
	(9.6×10^1^)	(46)		(8.4×10^1^)	(39)	
Chlorpyrifos	3.8×10^1^	290	4.3×10^5^	2.3×10^1^	220	1.0×10^6^
	(2.6×10^0^)	(27)		(2.1×10^0^)	(13)	

Standard deviations for the *k*_cat_ and *K*_M_ values are given in parentheses below the mean values obtained for triplicate experiments.

### Crystallographic analysis of PTE_Ar_ Ser308Leu/Tyr309Ala

In order to rationalise the effects of these mutations, we solved the crystal structure of the PTE*_Ar_* Ser308Leu/Tyr309Ala mutant (crystallographic data in Table 2). This did not reveal any significant changes to the backbone or B-factors of the loop that these mutations are located on (Loop 7). However, the Tyr309Ala mutation did expand the size of the active site entrance substantially (Fig. 3D). To investigate whether the enzyme, as constrained by the crystal packing, could still hydrolyze malathion, we performed a crystal soaking experiment, in which we soaked the crystal in 1 mM malathion for two minutes. The structural model obtained from the soaked crystal revealed density in the active site matching the product diethyl thiophosphate, in a known product binding mode [17]. This establishes that the crystal structure observed here is capable of hydrolysing malathion at rapid rates.

**Table 2:** Data collection and refinement statistics for structures reported in this work.

	PTE*_Ar_* Ser308Leu/Tyr309Ala	PTE*_Ar_*+2 mM malathion Ser308Leu/Tyr309Ala
Space group	P3 12 1	P3 12 1
Unit-cell parameters		
a (Å)	57.09	109.10
b (Å)	101.80	109.10
c (Å)	79.89	63.43
α,β,γ (**°**)	90, 90, 120	90, 90, 120
**Data collection**		
Wavelength (Å)	1.5418	1.5418
Resolution range (Å)*	29.36–1.99 (2.04–1.99)	28.22–1.99 (2.04–1.99)
No. of unique reflections	29386	30260
Redundancy	10.8 (9.9)	7.2 (6.6)
Completeness (%)	99.8 (97.8)	99.8 (98.4)
* R*_merge(I)_ †	0.122 (0.750)	0.123 (0.889)
Mean <*I*/σ(*I*)>	20.2 (4.0)	15.4 (2.4)
CC_1/2_^♯^	(0.998) (0.877)	0.997 (0.734)
**Refinement**		
No. reflections (total)	27872	28317
Resolution range	29.36–1.99 (2.04–1.99)	28.22–1.99(2.04–1.99)
* R*_work_/*R*_free_ ‡	0.158/0.186 (0.212/0.278)	0.206/0.251 (0.266–0.319)
**R.m.s deviations**		
Bond lengths (Å)	0.022	0.019
Bond angles (°)	2.084	2.036
**PDB ID**	3WML	4NP7

^*^Values in parenthesis are for the highest-resolution shell.

† *R*_merge(I)_  =  (Σ*_hkl_* Σ*_j_* |*I_hkl,j_−*〈*I_hkl_*〉|)/(Σ*_hkl_* Σ*_j_ I_hkl,j_*) where 〈*I_hkl_*〉 is the average intensity of *j* symmetry-related observations of reflections with Miller indices *hkl*.

# CC_1/2_  =  percentage of correlation between intensities from random half-datasets.

‡ *R*_work_  =  Σ*_hkl_*|F_(obs)_−F_(calc)_|/Σ*_hkl_*|F_(obs)_|; 5% of the data that were excluded from the refinement were used to calculate *R*_free_.

To investigate whether this increase in activity was a result of relieving the steric hindrance that was inhibiting substrate turnover in the wild-type enzyme, we performed substrate docking with malathion and the engineered Ser308Leu/Tyr309Ala variant (Fig. 3D). These results confirmed that the widening of the active site that occurs as a result of these mutations allows productive substrate binding and vastly improved turnover rates. It also explains the reduced turnover of paraoxon (Table 1): previous work has established that the interaction between the aromatic group of Tyr309 and the aromatic group of paraoxon, which is lost in this mutant, enhances catalysis.

## Summary

PTE*_Ar_* has potential in a wide range of applications, due its high turnover rates and broad substrate specificity. However, the applicability of the wild-type enzyme is limited for some substrates, such as malathion. Here we have enhanced the turnover rate for malathion by ∼5,000 fold using a semi-rational approach, which has alleviated the steric hindrance responsible for the low rate of malathion turn-over in the wild-type enzyme. The requirement for two amino acid substitutions, adjacent to each other in the protein, suggests that it would have been unlikely to have produced this specific variant by another, purely random, method.
